# Methanolic Extract of Ceplukan Leaf (*Physalis minima* L.) Attenuates Ventricular Fibrosis through Inhibition of TNF-*α* in Ovariectomized Rats

**DOI:** 10.1155/2016/2428052

**Published:** 2016-01-31

**Authors:** Bayu Lestari, Nur Permatasari, Mohammad Saifur Rohman

**Affiliations:** ^1^Biomedical Sciences, Medical Faculty, Brawijaya University, Malang 65145, Indonesia; ^2^Department of Pharmacology, Medical Faculty, Brawijaya University, Malang 65145, Indonesia; ^3^Department of Cardiology and Vascular Medicine, Medical Faculty, Brawijaya University, Saiful Anwar General Hospital, Malang 65145, Indonesia

## Abstract

The increase of heart failure prevalence on menopausal women was correlated with the decrease of estrogen level. The aim of this study is to investigate the effects of ceplukan leaf (*Physalis minima* L.), which contains phytoestrogen physalin and withanolides, on ventricular TNF-*α* level and fibrosis in ovariectomized rats. Wistar rats were divided into six groups (control (—); OVX 5: 5-week ovariectomy (OVX); OVX 9: 9-week ovariectomy; treatments I, II, and III: 9-weeks OVX + 4-week ceplukan leaf's methanolic extract doses 500, 1500, and 2500 mg/kgBW, resp.). TNF-*α* levels were measured with ELISA. Fibrosis was counted as blue colored tissues percentage using Masson's Trichrome staining. This study showed that prolonged hypoestrogen increases ventricular fibrosis (*p* < 0.05). Ceplukan leaf treatment also resulted in a decrease of ventricular fibrosis and TNF-*α* level in dose dependent manner compared to without treatment group (*p* < 0.05). Furthermore, the TNF-*α* level was normalized in 2500 mg/kgBW* Physalis minima* L. (*p* < 0.05) treatment. The reduction of fibrosis positively correlated with TNF-*α* level (*p* < 0.05, *r* = 0.873). Methanolic extract of ceplukan leaf decreases ventricular fibrosis through the inhibition of ventricular TNF-*α* level in ovariectomized rats.

## 1. Introduction

Incidences of heart failure increase on postmenopausal women related to hypoestrogen condition [1, 2]. Previous study also showed an increase in inflammatory response and myocardial fibrosis in an animal model of menopause, at least in part, via TNF-*α* pathway [2–4]. Estrogen possessed anti-inflammatory properties through transcription rate inhibition of several proinflammatory cytokines and cardioprotective effects [4].

Phytoestrogen is a group of substances originated from plants which have similar structure and functionality with estrogen [5]. The aim of this study is to investigate the effect of ceplukan leaf's methanolic extract (*Physalis minima* L.), which contains phytoestrogen physalin and withanolides [6–8], on ventricular TNF-*α* level and fibrosis in ovariectomized Wistar rats.

## 2. Materials and Methods

### 2.1. Animals

Three-month female Wistar rats (*Rattus norvegicus*) were kept in cages made of a plastic with a lid made of woven wire cage, with a cycle of 12 hours light/dark, fed, and watered by ad libitum. After 7 days of acclimatization, Wistar rats were divided into six groups (K1: normal; K2: 5-week ovariectomy (OVX); K3: 9-week ovariectomy (OVX), K4, K5, and K6: 9-week OVX + 4-week ceplukan leaf's methanolic extract doses 500, 1500, and 2500 mg/kgBW, resp.). The dosage of methanolic extract of ceplukan leaf was determined based on preliminary study (unpublished data). All procedures were approved by Health Research Ethics Committee of Brawijaya University.

### 2.2. Sample Preparation

Ceplukan leaves were obtained from* Balai Tanaman Obat Materia Medica*, Batu, Indonesia. Ceplukan leaves (dry powder) were weighed and wrapped in filter paper, inserted in funnel extraction, and then soaked with methanol to obtain the compounds. The solution from immersion process was then collected and precipitated. The solution was separated and collected from the precipitated product and then dried in rotator evaporator at 70–80°C to obtain the thick extract. The product was then heated in oven at 70°C to remove the remaining methanol.

### 2.3. Ovariectomy Procedure

Ovariectomy procedure was performed as previously described [9]. The rats were anaesthetized by intraperitoneal (IP) injection of ketamine (40 mg/kg). Ventral hair was shaved approximately 1 cm above the imaginary line ovaries. The site was then cleaned with povidone-iodine and alcohol 70%. The paralumbar lateral incision was made using a sharp knife and the ovaries were removed. The wound was sutured using catgut and covered by sterile gauze. Each rat was injected with gentamicin (60–80 mg/kg, IM) and cleaned with povidone iodine for 3 days after surgery to prevent postoperative infection.

### 2.4. Heart Morphometric Measurements

The separated heart organ from the pulmonary artery, pulmonary vein, aorta, and vena cava was cleaned from blood using saline solution. Heart organ was continuously cleaned optimally from vein and artery. An atrial part was separated from the whole heart organ carefully. Mitral and tricuspid valve was left for ventricular weight measurements. Right ventricle was separated carefully from the left ventricle. Septum interventricular was left for left ventricular measurements. Atrial weight, right ventricular weight, and left ventricular weight were measured using the digital analytic weighing machine at Pharmacology Laboratory, Faculty of Medicine, Brawijaya University.

### 2.5. TNF-*α* Level Measurements

TNF-*α* level was measured as previously described [10]. Briefly, heart ventricle was taken off approximately 100 mg and washed with distilled water and 1 mL PBS. The tissue protein was extracted by homogenizing the tissue in lysis buffer PMSF (containing Tris base, 0.1211 g; EDTA, 0.0074 g; NaCl, 0.8775 g; PMSF, 0.009 g; NP 40, 0.125 mL; deionized water, 100 mL; protease inhibitor cocktail, 50 *μ*L) for about 2 minutes. The mixture was then incubated for 30 minutes at 4°C and cold centrifuged at 6000 rpm for 10 minutes. The supernatant was taken to measure TNF-*α* levels using Quantikine Rat TNF-*α* kit (R&D Systems, USA, and Canada) according to the manufacturer instructions.

### 2.6. Ventricular Fibrosis Measurements

Ventricular fibrosis was measured as previously described [11]. Briefly, left ventricle that had been separated from the whole heart organ and analyzed for morphometric measurements was fixed in formalin 10% solution. After the fixation process for at least 1 day, ventricular tissue was blocked in paraffin and then sliced for histologic preparation using microtome. Histologic preparations of left ventricular tissue were stained with Masson's Trichrome. Ventricular fibrosis percentage was measured by counting the percentage of blue colored cells using software ImageJ. Fibrosis percentage was measured in 3 fields of view (40x ocular magnification) randomly at the midmyocardium area. This method was done to avoid both large artery and vein at epicardium area and artifact caused by compression/slicing process. Histologic preparation had been conducted at Pathologic Anatomy Laboratory, Faculty of Medicine, Brawijaya University.

### 2.7. Statistical Analysis

The results were expressed as means ± SD. Multiple comparisons were analyzed by one-way analysis of variance (ANOVA) followed by Tukey as post hoc test. The relationship between the two variables was examined using Pearson's correlation method. The level of significance was *p* < 0.05. All statistical tests were performed by SPSS 17.00.

## 3. Results

### 3.1. Heart Morphometric

Heart weight, right and left ventricular weight, and atrial weight had been measured as shown in Table 1. There were no significant differences among six groups based on heart weight, right ventricular weight, and atrial weight. Interestingly, this study showed significant differences of left ventricular weight between groups (ANOVA, *p* = 0.026). Furthermore, post hoc test revealed a significant increase of left ventricular weight (LVW) in 5-week ovariectomized rats (OVX 5) treatment placebo as compared to rats without treatment (negative control), suggesting that, after 5 weeks, ovariectomy procedure successfully induced hypertrophy in left ventricular. However, left ventricular hypertrophy in 9-week ovariectomized rats did not show any statistical differences compared to negative control or 5-week ovariectomized rats treatment. Treatment with 500 mg/kg body weight of ceplukan leaf' methanolic extract (treatment I) significantly decreased LVW similar to control (—). However, there was also no significant decrease of LVW in 1500 and 2500 mg/kgBW treated rats (treatment II and treatment III) compared to ovariectomized rats.

### 3.2. Ventricular TNF-*α* Level

**Figure 1:**
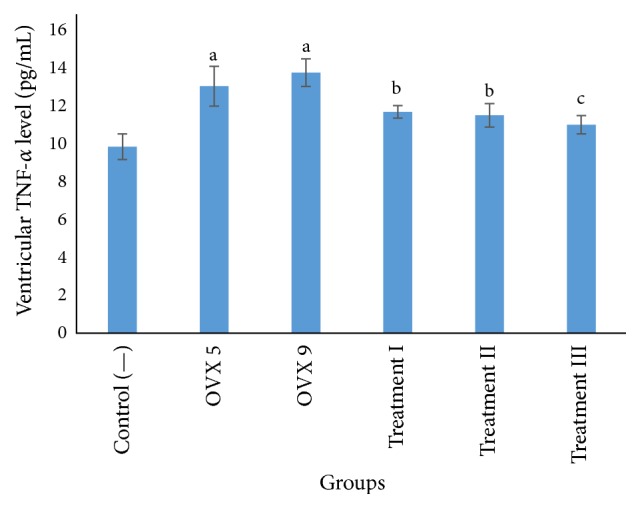
Mean of ventricular TNF-*α* level in each group (pg/mL). ^a^ *p* < 0.05 compared to control (—) group and treatment groups; ^b^ *p* < 0.05 compared to control (—) and OVX groups; ^c^ *p* < 0.05 compared to OVX groups. OVX 5: 5-week ovariectomy; OVX 9: 9-week ovariectomy. Treatments I, II, and III reflected 9-week ovariectomy treated with 500, 1500, and 2500 mg/kgBW ceplukan leaf's methanolic extract, respectively.

Ventricular TNF-*α* level of each group had been measured as shown in Figure 1. Ovariectomy procedure of both 5 weeks (OVX 5) and 9 weeks (OVX 9) significantly increased ventricular TNF-*α* level compared to negative control. However, there were no significant differences of ventricular TNF-*α* level between 5-week and 9-week ovariectomized rats. Treatment with either 500 or 1500 mg/kgBW (treatment I and treatment II) methanolic extract of ceplukan leaves successfully decreased ventricular TNF-*α* level compared to placebo treated ovariectomized rats. Interestingly, ventricular TNF-*α* level was normalized in 2500 mg/kgBW ceplukan leaf's methanolic extract treated rats. Pearson correlation test showed a strong negative correlation between the dose of ceplukan leaf's methanolic extract and ventricular TNF-*α* level (*p* = 0.000, *r* = −0.888).

### 3.3. Ventricular Fibrosis

**Figure 2:**
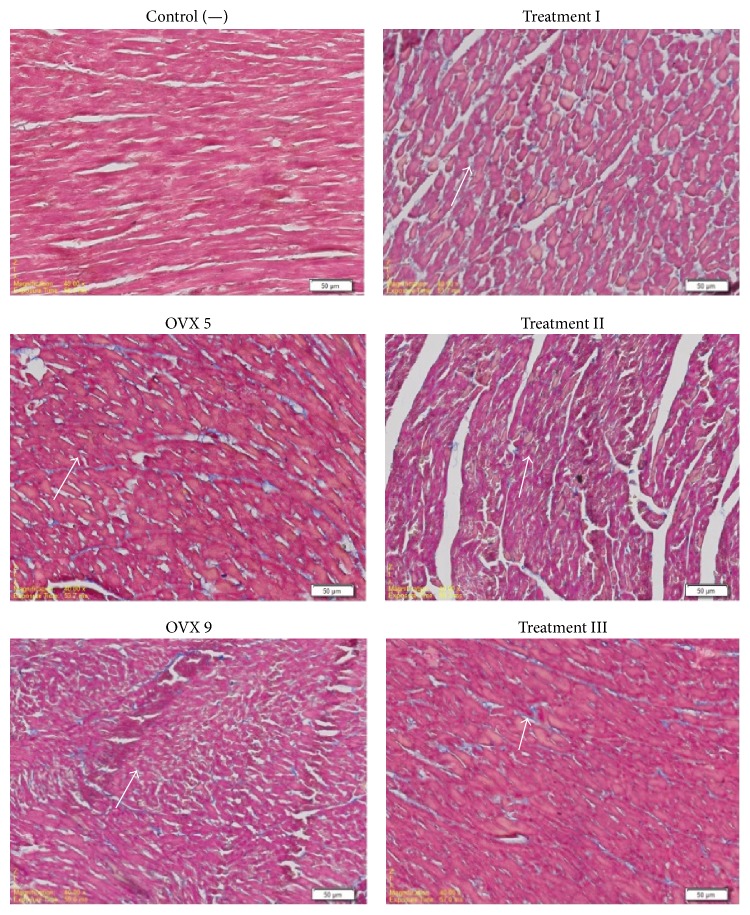
Masson's Trichrome staining of ventricular histologic preparation (ocular magnification 4x). Blue color indicates ventricular fibrosis (marked with white arrows).

**Figure 3:**
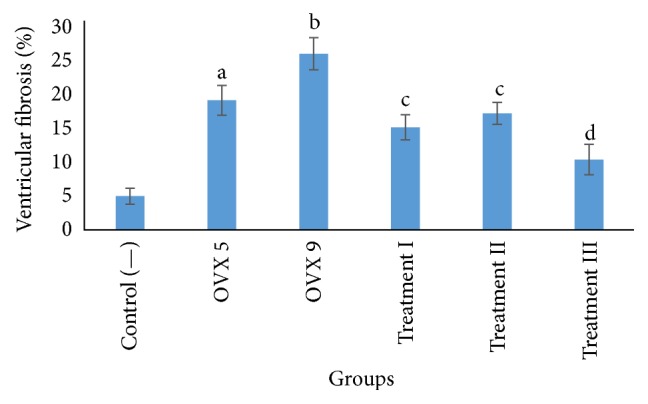
Mean of ventricular fibrosis in each group (%). ^a^ *p* < 0.05 compared to control (—), OVX 9, treatment III groups; ^b^ *p* < 0.05 compared to all other groups; ^c^ *p* < 0.05 compared to control (—) and OVX 9; ^d^ *p* < 0.05 compared to OVX and control (—). OVX 5: 5-week ovariectomy; OVX 9: 9-week ovariectomy. Treatments I, II, and III reflected 9-week ovariectomy treated with 500, 1500, and 2500 mg/kgBW ceplukan leaf's methanolic extract, respectively.

Ventricular fibrosis presented in blue colored tissues using Masson's Trichrome staining shown in Figure 2. Semiquantitative measurements of ventricular fibrosis using ImageJ were done as shown in Figure 3. Ovariectomy procedure of both 5 weeks (OVX 5) and 9 weeks (OVX 9) significantly increases ventricular fibrosis, suggesting that hypoestrogenic condition induces fibrosis formation of ventricular tissue. Furthermore, 9-week ovariectomy procedure significantly increases ventricular fibrosis compared to 5-week ovariectomy procedure. All variant dose resulted in a significant decrease of ventricular fibrosis compared to 9-week ovariectomized rats. However, ventricular fibrosis was not normalized even with the highest dose of 2500 mg/kgBW ceplukan leaf's methanolic extract. Pearson correlation test showed a strong negative correlation between the dose of ceplukan leaf's methanolic extract and ventricular fibrosis percentage (*p* = 0.000, *r* = −0.860).

### 3.4. Correlation of Ventricular TNF-*α* Level and Fibrosis

Pearson's correlation test showed a strong positive correlation between ventricular TNF-*α* level and fibrosis (*p* = 0.000, *r* = 0.873). This result suggested that there was a causative correlation between inflammation (particularly TNF-*α* level) and fibrosis formation in ventricular tissue.

## 4. Discussion

### 4.1. Ovariectomy and Left Ventricular Hypertrophy, TNF-*α* Level, and Fibrosis

This study showed that left ventricular weight was elevated in 5-week ovariectomized rats compared to negative control. Ovariectomy-induced hypoestrogenic state also resulted in the elevation of TNF-*α* in 5-week and 9-week ovariectomized rats. The results in accordance with the previous study reported about the correlation of depleted estrogen (caused by either ovariectomy or knockout of ER*β*) and cardiac hypertrophy [12–14]. Hypertrophied myocardium is usually accompanied with interstitial fibrosis which is characterized by the increase of collagen genes expression [15] which could affect coordinated excitation-contraction coupling of cardiomyocytes and induce diastolic stiffness and impairing cardiac output [16, 17].

Moreover, ovariectomized rats model also showed an elevated secretion of various proinflammatory cytokines such as TNF-*α* in hypoestrogenic state and animal model of menopause [2, 18, 19]. As previously studied, there was a correlation between estrogen depletion and inflammation marked by elevated TNF-*α* level [20, 21].

There was also elevated ventricular fibrosis in 5-week and 9-week ovariectomized rats compared to negative control group. Moreover, in 9-week ovariectomized rats treatment, there was a significant elevated fibrosis compared to 5-week ovariectomized rats. Interestingly, further increase of fibrosis in 9-week ovariectomized rats was not in line with further increase of TNF-*α*. These results indicate that inflammation alone did not cause ventricular fibrosis. Previous study showed that ovariectomy without estrogen replacement was associated with elevated expression of proapoptotic, proinflammatory, and profibrotic genes [22].

Cardiac fibrosis formation in ovariectomized rats could be triggered by myocardial cell apoptosis (both intrinsic and extrinsic) [4] and angiotensin II-induced fibrosis [23]. Extrinsic apoptosis pathway was induced by TNF-*α* as a ligand which in turn leads to heart remodeling marked by fibrosis [24, 25]. Otherwise, angiotensin II could increase TNF expression regulation through NF-*κ*B (nuclear factor kappa B) dependent pathway [24–26]. ER-*β* knockout on mice showed an elevated transcription of cardiac proapoptotic genes [13]. In hypertensive ovariectomized rat model, hypoestrogenic condition is able to augment cardiac inflammation and oxidative stress and it thus aggravates myocardial fibrosis and diastolic dysfunction [27].

In accordance with the previous study, the results showed that TNF-*α* strongly correlated with fibrosis, suggesting that inflammation process triggered fibrosis formation and is macroscopically represented by left ventricular hypertrophy. However, this study needs further investigation on the impact of hypoestrogenic duration on ventricular inflammation, apoptosis, RAAS, fibrosis, and also left ventricular functionality.

### 4.2. Ceplukan Leaf's Methanolic Extract Treatment and Left Ventricular Hypertrophy, TNF-*α* Level, and Fibrosis

Gilles and colleagues [28] reported that ceplukan leaf contains 13,14-seco-16,24-cyclosteroid called physalin. Physalin consists of several compounds such as physalin A, physalin C, physalin D, 5*β*-6*β* epoxyphysalin, dihydroxyphysalin B, whitaphysalin A, whitaphysalin B, and whitaphysalin C [5]. Besides physalin,* Physalis minima* L. also contains withanolides, molecule that also possessed estrogenic activity and had been studied for its antifibrotic activities [7, 8].

Treatment with 500 mg/kgBW of ceplukan leaf's methanolic extract significantly decreases left ventricular hypertrophy compared to 5-week ovariectomized rats. The effect of estrogen and phytoestrogen on heart morphometric revealed controversy results. Previous study demonstrated that estradiol administration did not significantly affect heart morphometric compared with 3.5-week ovariectomized rats [19]. Conversely, Tang and colleagues reported that estradiol administration significantly decreases heart weight and heart weight/body weight ratio compared to ovariectomized rats, but phytoestrogen genistein did not show similar results [29]. In chronic volume overload model, either estradiol or phytoestrogen administration decreased ventricular remodeling [30, 31].

Estrogen via ER-*β* has been found to attenuate cardiac hypertrophy [32]. Several mechanisms have been proposed to explain the effect of estrogen administration on cardiac hypertrophy such as the mitigation of Ang-II signaling [33] and increased degradation of calcineurin as hypertrophic factor [34].

Treatment with ceplukan leaf's methanolic extract also significantly decreases TNF-*α* level and was normalized in the highest dose treatment (2500 mg/kgBW). Furthermore, the decreased ventricular TNF-*α* level evidently inhibits ventricular fibrosis in ceplukan leaf's methanolic extract treated rats. Interestingly, normalized TNF-*α* level in 2500 mg/kgBW ceplukan leaf's methanolic extract was not followed by normalized fibrosis, suggesting that fibrosis signaling was not independently caused by TNF-*α*.

Estrogen possessed anti-inflammation properties through the downregulation of several proinflammatory cytokines such as TNF-*α* [20, 35, 36] and cardioprotective effects through the upregulation of eNOS (endothelium-derived nitric oxide) [37] and cardiac biopterins [38]. Previous study also reported that estradiol binds to ER-*β* which inhibits TNF-*α* expression in rat's aortic smooth muscle cell culture via dependent pathway [39] and heart fibrosis pathway through the inhibition of downstream activity caused by Angiotensin II-induced TGF-*β* (Transforming Growth Factor-*β*) [40]. Angiotensin II is an important molecule in neonatal cardiac fibroblast proliferation [40, 41] and collagen deposition [41] and this process could be inhibited by estradiol administration [40–42].

The effect of phytoestrogen administration on ventricular inflammation and fibrosis had been previously studied using genistein. Genistein administration in human umbilical vascular endothelial cells (HUVECs) culture decreased monocyte adhesion induced by TNF-*α* [43]. Another study reported that the combination of herbs which contains withanolides showed antiapoptotic and cardioprotective effects in acute myocardial infarct model [8].

Our study showed a positive correlation between cardiac inflammation and fibrosis, and we hypothesized that methanolic extract of* Physalis minima* which contains phytoestrogen could inhibit cardiac inflammation, thereby inhibiting cardiac fibrosis. This finding was in accordance with the previous study which reported that proinflammatory cytokines are critically involved in modulating the initial myocardial remodeling suggesting that a reduction or prevention of an inflammatory response by phytoestrogenic compounds attenuates the development of adverse ventricular dilatation [44].

However, this study did not investigate the effects of ceplukan leaf's methanolic extract on myocardial apoptosis. This pathway should be confirmed with more advanced research. Furthermore, fibrosis pathway is not only triggered by TNF-*α*, but also triggered by several pathways such as TGF-*β* signaling that in turn induced various intracellular protein kinase signaling for fibrosis formation. To investigate this pathway, further studies are needed.

## 5. Conclusion

We concluded that ceplukan leaf's methanolic extract could decrease the ventricular fibrosis through the inhibition of TNF-*α* in ovariectomized rats. The duration of hypoestrogen increases ventricular fibrosis but not TNF-*α* level.

## Figures and Tables

**Table 1:** Measurements of heart morphometric.

Parameters	Control (—)	OVX 5	OVX 9	Treatment I	Treatment II	Treatment III	ANOVA
BW (gram)	236.5 ± 14.2	280.8 ± 33.4	264.3 ± 20.0	237.0 ± 19.9	250.8 ± 16.5	252.5 ± 34.6	0.124^*∗*^
LVW (mg)	501 ± 16.2	578.8 ± 54.6^#^	547 ± 41.6	502.6 ± 25.1	519.2 ± 40.3	514.5 ± 30.8	0.026^*∗∗*^
RVW (mg)	127 ± 15.6	136.6 ± 22.0	119.7 ± 16.9	125.4 ± 24.9	121.8 ± 16.9	119.3 ± 8.3	0.706
AW (mg)	82.9 ± 3.4	88.9 ± 10.0	81.5 ± 5.05	88.8 ± 2.9	84.4 ± 5.5	80.3 ± 1.7	0.174
LVW/HW ratio	0.705	0.720	0.731	0.704	0.716	0.720	0.298
HW/BW ratio	3.01 × 10^−3^	2.87 × 10^−3^	2.85 × 10^−3^	3.03 × 10^−3^	2.90 × 10^−3^	2.85 × 10^−3^	0.757

^*∗*^Statistical analysis for body weight using Kruskal-Wallis test as post hoc test. ^*∗∗*^ *p* < 0.05 using one-way ANOVA analysis. ^#^ *p* < 0.05 compared with control (—) using Tukey's test as post hoc test.

BW: body weight; LVW: left ventricular weight; RVW: right ventricular weight; AW: atrial weight; OVX 5: 5-week ovariectomy; OVX 9: 9-week ovariectomy; Treatments I, II, and III: 9-week ovariectomy with 500, 1500, and 2500 mg/kgBW ceplukan leaf's methanolic extract, respectively.
